# The Pathology and Surgical Treatment of Tumors

**Published:** 1897-09

**Authors:** 


**The Pathology and Surgical Treatment of Tumors.** By N. Senn, M.D. Pp. 709. Philadelphia: W. B. Saunders. 1895.

Professor Senn's book on tumours, which is a little late in reaching us, will be received with much interest and pleasure by the profession both in America and other countries. It is a voluminous treatise, the result of a collection of material extending over many years, and of much laborious work. The chapters on the pathology of tumours at the commencement of the book are very valuable, and thoroughly interesting. Unfortunately too much has been attempted in the preparation of this vast compendium. In dealing with the subject of diagnosis and treatment an attempt has been made to cover far too much ground. For instance, the writer has attempted to give, in connection with the treatment of malignant growths of the stomach and intestines, and of tumours of the uterus and ovaries, what would be much more in place in a large work on abdominal surgery. The information given about operative treatment is so insufficient as to be practically useless, but may unfortunately mislead some by allowing them to suppose that when they have read Senn's account they have obtained all the necessary information. The same incompleteness is conspicuous in the part devoted to the diagnosis of tumours of the breast, and in the account of excision of the breast for cancer no mention is made of the necessity of removing the portion of skin which lies over the scirrhous growth in whatever portion of the breast the growth may be situated. We are told that in scirrhus of the breast pain of a shooting and lancinating character is always present after the tumour has attained a certain size, and yet we know that more often pain is completely absent for many months after the accidental discovery of the growth. If the author intended to imply that pain was always present late in the disease he should have made his meaning quite clear, for the idea that early scirrhus is always painful would lead to frequent failure in the diagnosis of cancer in that stage when its treatment is the most hopeful. Moreover, the prognosis of cancer of the breast which is given by Dr. Senn is not that which the more thorough modern operation has made possible in the practice of several surgeons.

It would have been much better, we venture to think, if the writer had limited his subject to the pathology of tumours. We are glad to see he rejects the microbic origin of true tumours as not yet proved, and that he separates retention cysts from tumours proper. He adheres to Cohnheim's view of the embryonic origin of tumours, but we have looked in vain in this work for a statement of the evidence on which Cohnheim and the author found their belief. Dr. Senn considers that there may be what he calls "post-natal embryonic cells," or embryonic cells the offspring of mature cells which, for some reason, have failed to undergo transformation into tissue of a higher type, and which may remain in a latent immature state for an indefinite period, to become, under the influence of either hereditary or acquired exciting causes, the essential starting point of a tumour. What he means by this is, that the new cells produced in repair may remain undeveloped into mature tissue and later on grow into a tumour. It seems to be rather from the absence of better proof of other origins that the author accepts Cohnheim's theory and endeavours to formulate another which he appends to Cohnheim's, than from the strength of any arguments in its favour. Dr. Senn reports (p. 86) a case of a patient who developed myeloid sarcoma at the seat of a fracture, he believes from callus, and argues that as there had been no pain before the fracture the growth must have been secondary. This view we fail to accept, for we have observed the almost painless growth of myeloid sarcoma. We consider that it was quite as likely—indeed more likely—that fracture resulted from the presence of the growth. The statement (on p. 21) that tumours never disappear except by removal or destruction, is a little too absolute in the light of some recent well authenticated cases. It would, we consider, have been more correct had the author stated that such an occurrence was of the greatest rarity, and that most cases in which swellings diagnosed as tumours disappeared were really infective or simple granulomata, and not tumours in the true sense of the word. The chapter on transformation of innocent into malignant tumours is an interesting one, but might have been enriched with more modern cases which are supposed to support the opinion that such transference does occur. The transformation of fibro-myoma of the uterus into sarcoma is accepted as a fact. We have been much disappointed in turning to the chapter on lymphatic growths not to find the difficult question as to the exact relationship between sarcoma and lymphadenoma more fully considered.

The work is one well worth careful study, and will be found useful for frequent reference. It is profusely illustrated. Many illustrations are evidently from micro-photographs, and hence some of them lack definition of structure. However, they possess perfect accuracy of representation, so far as it goes. Many pictures and much information have been taken from Bland Sutton's work on tumours, and acknowledged as so derived.

